# Species-Specific Inhibition of Necroptosis by HCMV UL36

**DOI:** 10.3390/v13112134

**Published:** 2021-10-22

**Authors:** Elena Muscolino, Claudia Castiglioni, Renke Brixel, Giada Frascaroli, Wolfram Brune

**Affiliations:** 1 Leibniz Institute for Experimental Virology (HPI), 20251 Hamburg, Germany; elena.muscolino@upf.edu (E.M.); claudia.castiglioni3@studenti.unimi.it (C.C.); renke.brixel@leibniz-hpi.de (R.B.); giada.frascaroli@leibniz-hpi.de (G.F.); 2 Molecular Virology Group, Department of Experimental and Health Sciences, Universitat Pompeu Fabra, 08003 Barcelona, Spain

**Keywords:** apoptosis, necroptosis, RIPK1, RIPK3, MLKL, UL36, M45, ICP6, HCMV, MCMV, herpesvirus

## Abstract

Viral infection activates cellular antiviral defenses including programmed cell death (PCD). Many viruses, particularly those of the *Herpesviridae* family, encode cell death inhibitors that antagonize different forms of PCD. While some viral inhibitors are broadly active in cells of different species, others have species-specific functions, probably reflecting the co-evolution of the herpesviruses with their respective hosts. Human cytomegalovirus (HCMV) protein UL36 is a dual cell death pathway inhibitor. It blocks death receptor-dependent apoptosis by inhibiting caspase-8 activation, and necroptosis by binding to the mixed lineage kinase domain-like (MLKL) protein and inducing its degradation. While UL36 has been shown to inhibit apoptosis in human and murine cells, the specificity of its necroptosis-inhibiting function has not been investigated. Here we show that UL36 interacts with both human and murine MLKL, but has a higher affinity for human MLKL. When expressed by a recombinant mouse cytomegalovirus (MCMV), UL36 caused a modest reduction of murine MLKL levels but did not inhibit necroptosis in murine cells. These data suggest that UL36 inhibits necroptosis, but not apoptosis, in a species-specific manner, similar to ICP6 of herpes simplex virus type 1 and MC159 of molluscum contagiosum virus. Species-specific necroptosis inhibition might contribute to the narrow host range of these viruses.

## 1. Introduction

Herpesviruses are important human pathogens widely spread among the human population. They cause long-life infections characterized by phases of latency and reactivation [1,2]. After virus entry, the host cell recognizes the infection and triggers innate immune responses that lead to the production of inflammatory cytokines, type I interferon (IFN), and various molecules that activate the adaptive immune response [3]. Next to this, cellular defense mechanisms that restrict viral replication are activated, such as the shutoff of protein translation, the degradation of viral components via the proteasome or lysosome pathways, and the induction of cell death [4,5,6].

Programmed cell death (PCD) plays a key role as a defense mechanism against virus infection. Mammalian cells can activate different signal transduction pathways that ultimately lead to their death, including intrinsic and extrinsic apoptosis, necroptosis, pyroptosis, autophagy-dependent cell death, and a few others [7]. PCD of infected cells can be beneficial for the host as it serves to eliminate the intracellular niche of certain pathogens. Apoptosis and necroptosis, the two best-characterized forms of PCD, can limit viral infection and dissemination [8]. PCD can be activated upon binding of a cognate ligand to a specific cell surface receptor. This binding triggers a series of downstream events leading to the cleavage of several cellular proteins by intracellular enzymes (in particular caspases), in the case of apoptosis, or the release of cytosolic contents into the extracellular space by a caspase-independent mechanism, in the case of necroptosis [9]. Besides their morphological differences, extrinsic apoptosis and necroptosis can be triggered by the same class of receptors [10,11]. Activation of death receptors such as the tumor necrosis factor receptor 1 (TNFR1), or Fas leads to the assembly of TNFR Complex II and to the cleavage of caspase-8 that in turn initiates extrinsic apoptotic cell death [12]. When caspase-8 is inhibited, a pathway leading to necroptosis may be activated. Necroptosis depends on the two receptor interacting protein kinases 1 and 3 (RIPK1 and RIPK3) that interact through a RIP homotypic interaction motif (RHIM) domain causing a caspase-independent type of cell death [13]. Necroptosis can also be activated by Z-DNA-binding protein 1 (ZBP1) or by TIR-domain-containing adapter-inducing interferon-β (TRIF), an adapter protein of Toll-like receptors 3 or 4 (TLR3/4), which also contain a RHIM domain capable of activating RIPK3 [14]. In all three pathways, activated RIPK3 phosphorylates the mixed lineage kinase domain-like protein (MLKL) leading to its oligomerization [15]. MLKL oligomers translocate to the plasma membrane, where they form pores or channels, which alter membrane permeability and lead to cell swelling and cell death [14].

**Figure 1:**
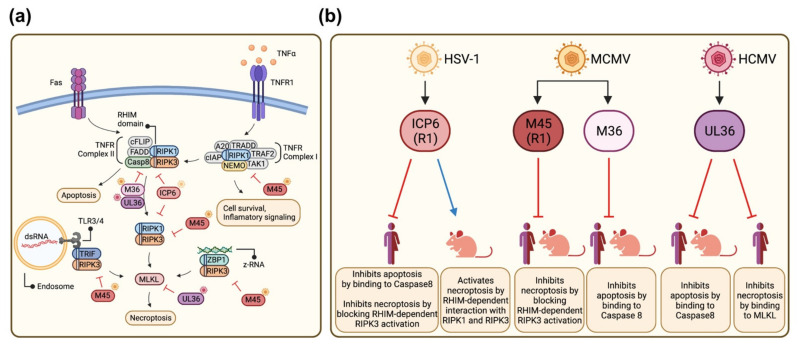
Inhibition of apoptosis and necroptosis by HSV-1, HCMV, and MCMV. (**a**) Schematic representation of death receptor-dependent (extrinsic) apoptosis and necroptosis signaling pathways. The viral inhibitors (marked with virus symbols) and their targets are shown. (**b**) Known functions of the viral cell death inhibitors in human and murine cells. Figure generated with BioRender.com (accessed on 21 October 2021).

Herpesviruses encode proteins that inhibit PCD to prevent premature cell demise and to provide more time for viral replication [16,17,18]. Herpes simplex virus type 1 (HSV-1) encodes for a viral ribonucleotide reductase (R1) homolog named ICP6 that acts as a dual cell death pathway inhibitor. It inhibits apoptosis by binding caspase-8 and blocks necroptosis by interacting with RIPK1 and RIPK3 at TNFR complex II in a RHIM-dependent manner [19,20,21,22] (Figure 1a). The human cytomegalovirus (HCMV) UL36 protein also functions as a dual cell death pathway inhibitor. Similar to ICP6, UL36 blocks caspase-8 activation to prevent apoptosis [23]. However, it blocks necroptosis by interacting with the key necroptosis mediator MLKL and inducing its degradation [24] (Figure 1a).

Interestingly, murine cytomegalovirus (MCMV), a mouse beta-herpesvirus, encodes two distinct proteins for the inhibition of the two PCD pathways. The MCMV R1 homolog, M45, blocks necroptosis by inhibiting RHIM-dependent activation of RIPK3, whereas M36, a homolog of UL36, blocks caspase-8 activation [25,26,27,28] (Figure 1a).

PCD inhibition by herpesviruses appears to be species-specific in some cases (Figure 1b). For instance, HSV-1 ICP6 inhibits necroptosis in human cells but stimulates necroptosis in mouse cells, suggesting that HSV-1 has specifically adapted its necroptosis inhibitor to its human host [19,20,22] (Figure 1b). By contrast, the MCMV homolog M45 prevents necroptosis in both human and murine cells [29]. Another example of a species-specific PCD inhibitor is the HCMV UL37x1 protein, also known as viral mitochondrion-localized inhibitor of apoptosis (vMIA). It prevents the execution of intrinsic apoptosis by targeting BAX and BAK, the two effector proteins of mitochondrial outer membrane permeabilization [30,31,32,33,34]. However, UL37x1 inhibits only BAX, but not BAK, in mouse cells [31,35]. For the same purpose, MCMV expresses two separate inhibitors: m38.5, a BAX-specific inhibitor similar to HCMV UL37x1 [36,37], and m41.1, a BAK-specific inhibitor [35,38].

Other viral PCD inhibitors are more broadly active. For instance, the MCMV M36 protein inhibits caspase-8 activation in murine as well as in human cells [28,39], and the same holds true for HCMV UL36. A recent study has demonstrated that a chimeric MCMV expressing HCMV UL36 instead of M36 retains the ability to inhibit caspase-8-dependent apoptosis in murine cells and infected mice [40]. However, the ability of UL36 to inhibit necroptosis in murine cells has not yet been investigated.

Herein we show that UL36 interacts with murine MLKL (mMLKL), whereas M36 does not. Compared to its interaction with human MLKL (hMLKL), the interaction with mMLKL is relatively weak. UL36 expression by a recombinant MCMV leads only to a modest reduction of mMLKL levels at late times post-infection and does not prevent necroptosis. We conclude that the ability of HCMV UL36 to inhibit MLKL is not conserved in MCMV M36. Moreover, UL36 inhibits necroptosis in human but not in murine cells, reflecting the high degree of adaptation of HCMV to its human host.

## 2. Materials and Methods

### 2.1. Cells and Viruses

NIH-3T3 (CRL-1658), SVEC4-10 (CRL-2181), HT-29 (HTB-38), were obtained from ATCC. 10.1 cells are immortalized mouse embryonic fibroblasts [41]. HEK-293A cells were purchased from Invitrogen (Karlsruhe, Germany). Cells were cultured at 37 °C and 5% CO_2_ in Dulbecco’s modified eagle medium (DMEM) medium supplemented with 5% or 10% fetal calf serum.

The repaired MCMV Smith strain BAC pSM3fr-MCK-2fl was kindly provided by Barbara Adler (University of Munich, Germany) [42]. MCMV mutants MCMVΔM45, MCMV-M45HA, and MCMV-M45*mut*RHIM have been described [43]. The MCMVΔM36 and MCMV-UL36 mutants were kindly provided by Luka Cicin-Sain (Helmholtz Centre for Infection Research, Braunschweig, Germany) [40]. MCMV-UL36 M45*mut*RHIM and MCMVΔM36 M45mutRHIM were generated by introducing point mutations within the M45 RHIM domain, amino acid sequence 61–64 (IQIG to AAAA), as previously described [26], into the MCMV-UL36 and MCMVΔM36 BACs by en passant mutagenesis [44]. WT and mutant MCMVs were grown and titrated on NIH-3T3 cells according to standard procedures [45]. Viral titers were determined using the median tissue culture infective dose (TCID_50_) method and infections were carried out with centrifugal enhancement (800× *g*, 30 min) when required [46].

### 2.2. Antibodies

Antibodies recognizing the following epitopes and proteins were used: HA (16B12, Covance, Münster, Germany), HA (3F10; Roche), Flag (F3165, Sigma, Taufkirchen, Germany), myc (4A6; Millipore, Darmstadt, Germany), IE1 (CROMA101; CapRi, Rijeka, Croatia), GAPDH (14C10; Cell Signaling, Frankfurt, Germany), β-Actin (AC-15; Sigma), mMLKL (AP14272B, Abgent, San Diego, CA). Mouse monoclonal antibodies against M45 and UL36 [47] were kindly provided by Stipan Jonjic (University of Rijeka, Croatia) and Thomas Shenk (Princeton University, USA), respectively. Secondary antibodies coupled to Alexa Fluor 488 (A-11034, A-11006, A-11029) and Alexa Fluor 555 (A-21424, A-21429, A-21434) were purchased from Invitrogen. Secondary antibodies coupled to horseradish peroxidase were purchased from DakoCytomation or Jackson ImmunoResearch.

### 2.3. Plasmids

pcDNA3 (Invitrogen), pcDNA3 Flag-IFI16 (Addgene, Watertown, MA), and pEPkan-S [44] were obtained from the indicated sources. pcDNA3 HA-UL36 was generated by inserting the PCR-amplified UL36 sequence between the HindIII and BamHI restriction sites of pcDNA3. pcDNA3 HA-M36 was generated in an analogous fashion. pcDNA3 Flag-hMLKL full length and truncation mutants were generated by inserting the PCR-amplified sequences between the EcoRI and XhoI sites of pcDNA3. pcDNA3 Flag-mMLKL full length and truncation mutants were generated by inserting the PCR-amplified sequences between the BamHI and XhoI restriction sites.

### 2.4. Transfection

HEK-293A cells were transfected by combining 8 μg of plasmid DNA and 32 μL of polyethylenimine transfection reagent (Sigma). Murine fibroblasts were transfected using Lipofectamine 2000 (ThermoFisher, Dreieich, Germany) according to the manufacturer’s protocol. For virus reconstitution, NIH-3T3 cells were transfected using 3 μg of BAC DNA and 10 μL Polyfect (Qiagen, Hilden, Germany).

### 2.5. Cell Viability Assay

Murine SVEC4-10 endothelial cells or 10.1 cells (5 × 10^3^) were seeded in 96-well plates and infected with MCMV at a MOI of 5 TCID_50_/cell in 100 µL media. Cell viability was determined at 24 h post infection (hpi) by measuring intracellular ATP levels with a Cell Titer-Glo Luminescent Cell Viability Assay kit (Promega, Madison, WI) and a FLUOstar Omega luminometer (BMG Labtech, Ortenberg, Germany). Cell were either treated 1 h prior to infection with cell death inhibitors Z-VAD-FMK (R&D Systems, Abingdon, UK), GSK’872 (Merck, Darmstadt, Germany), or DMSO (control) and remained on the cells for the duration of the assay, or were treated 6 hpi with 30 ng/mL TNF-α (R&D Systems), 1 µM BV6 (Selleckchem, Houston, TX, USA), 75 µM Z-VAD-FMK, GSK’872 (3 µM), and DMSO (as control) and remained on the cells for the duration of the assay. Significance was calculated using two-way ANOVA. Viability of human HT-29 cells was determined essentially as described [48]. Cells (5 × 10^3^) were seeded in 96-well plates and infected with MCMVs at an MOI of 5 PFU/cell in DMEM containing 1% FCS. Six hpi cells were treated with 30 ng/mL TNF-α, 1 µM BV6, 75 µM Z-VAD-FMK, 3 µM GSK’872, or DMSO (as control) were added. Cell viability was determined 24 hpi as described above.

### 2.6. Immunodetection

For immunoblot analysis of whole cell lysates, cells were lysed in boiling 2x SDS-PAGE sample buffer (125 mM Tris-HCl pH 6.8, 4% SDS, 20% glycerol, 10% β-mercaptoethanol). For immunoprecipitation, cells were lysed with lysis buffer containing 1% Nonidet P-40 (ThermoFisher) and cOmplete Mini Protease Inhibitor Cocktail (Roche, Penzberg, Germany). After pre-clearing, proteins of interest were precipitated by using specific antibodies combined with protein A or G Sepharose beads (GE Healthcare, Braunschweig, Germany). HA-tagged proteins were pulled-down with anti-HA affinity matrix (Roche). Precipitates were washed six times and then eluted by boiling in SDS-PAGE sample buffer. Samples were separated on denaturing SDS-PAGE gels and transferred electrophoretically onto nitrocellulose (GE Healthcare) by semi-dry blotting. Densitomerty was measured with NIH ImageJ [49].

Immunofluorescence staining and analysis was performed as described [43]. Fluorescence was detected by Nikon A1 confocal laser scanning microscope.

## 3. Results

### 3.1. HCMV UL36 Interacts with Human and Murine MLKL

**Figure 2:**
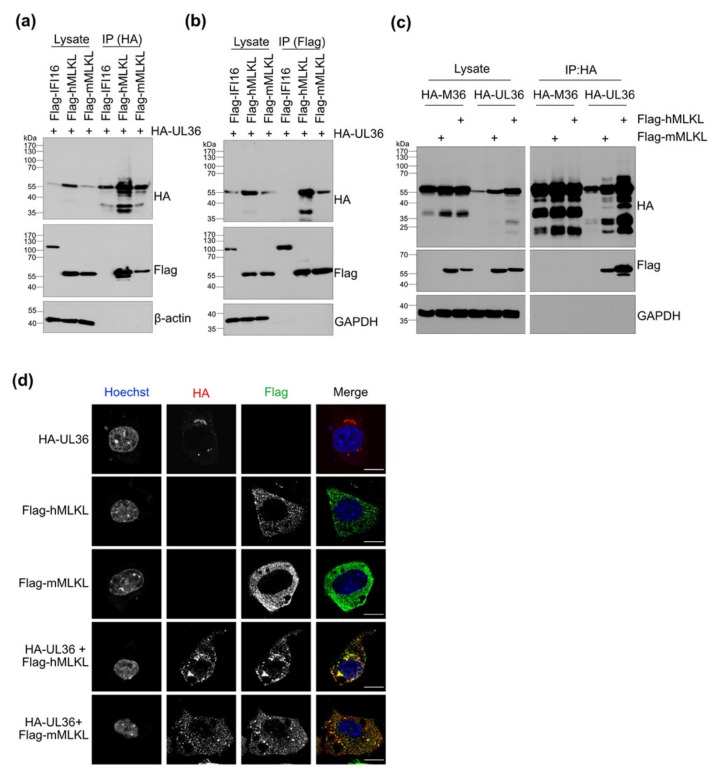
HCMV UL36 co-precipitates and co-localizes with human and murine MLKL. (**a**) HEK-293A cells were co-transfected with plasmids expressing HA-tagged UL36 and either Flag-tagged mMLKL or Flag-tagged hMLKL. HA-UL36 was immunoprecipitated, and co-precipitating Flag-tagged MLKLs were detected by Western blot. Flag-tagged IFI16 was used as a negative control. (**b**) HEK-293A cells were transfected with plasmids as described above. Flag-tagged proteins were immunoprecipitated, and co-precipitating HA-UL36 was detected by Western blot. (**c**) HEK-293A cells were co-transfected with plasmids expressing HA-tagged M36 or UL36 and Flag-tagged mMLKL or hMLKL. HA-tagged proteins were immunoprecipitated, and co-precipitating Flag-tagged MLKLs were detected by Western blot. (**d**) NIH-3T3 cells were transfected with plasmids encoding HA-UL36 (Red), and Flag-tagged hMLKL or mMLKL (Green). At 24 h post-transfection, the proteins were detected by immunofluorescence using tag-specific antibodies. Nuclei were stained with Hoechst 33342 (Blue). Scale bar, 10 µm.

The HCMV UL36 protein functions as a viral inhibitor of caspase-8-dependent apoptosis [23]. Recently, it has been shown that UL36 also inhibits necroptosis by interacting with hMLKL [24]. UL36 interacts with both human and murine caspase-8 and inhibits apoptosis in human and murine cells [23,40]. Thus, we wanted to test whether UL36 can also interact with both human and murine MLKL. For this purpose, HEK-293A cells were co-transfected with plasmids encoding Flag-tagged human or murine MLKL and HA-tagged UL36. HA or Flag-tagged proteins were immunoprecipitated, and co-precipitating proteins were detected by immunoblot. Under these conditions, hMLKL and mMLKL co-precipitated with UL36 and vice versa (Figure 2a,b).

Interestingly, we regularly observed higher UL36 protein levels in cells expressing hMLKL compared to those expressing mMLKL (Figure 2a,b). Additionally, more UL36 protein co-precipitated with hMLKL (Figure 2b), suggesting a stronger interaction of UL36 with hMLKL than with mMLKL.

To test whether M36, the MCMV homolog of HCMV UL36, is capable of interacting with MLKL, HEK-293A cells were transfected with plasmids encoding Flag-tagged mMLKL or hMLKL and HA-tagged M36 or UL36. HA-tagged proteins were immunoprecipitated. While both human and murine MLKL proteins co-precipitated with UL36, they did not co-precipitate with M36 (Figure 2c), suggesting that the interaction with MLKL is a property of UL36 not shared by M36.

Next, we tested by immunofluorescence whether UL36 and mMLKL co-localize in transfected cells. NIH-3T3 cells were transfected with plasmids expressing HA-UL36, and Flag-tagged mMLKL or hMLKL. HA-UL36 co-localized with both MLKLs proteins. However, the distribution of the MLKL proteins changed in the presence of UL36. While hMLKL and mMLKL were quite evenly distributed throughout the cytoplasm in the absence of UL36, hMLKL and (to a lesser extent) mMLKL accumulated in perinuclear clusters in the presence of UL36 (Figure 2d).

### 3.2. UL36 Interacts with the N-Terminal Domain of MLKLs

**Figure 3:**
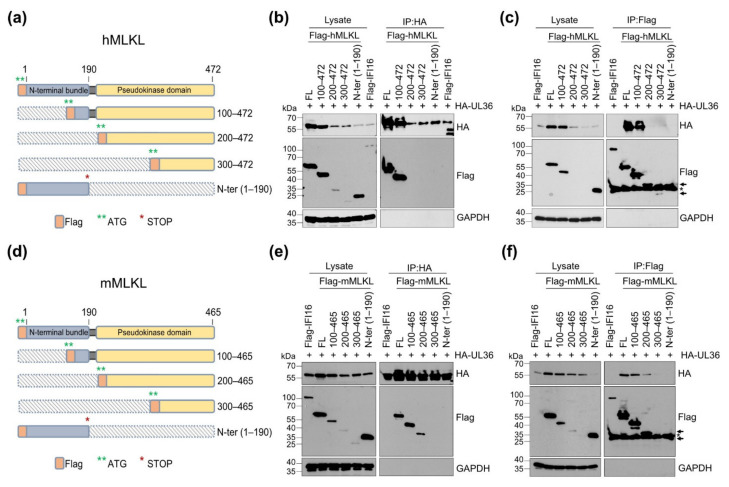
HCMV UL36 interacts with the N-terminal domain of MLKL. (**a**) Schematic depiction of hMLKL, consisting of an N-terminal four-helix bundle and a C-terminal pseudokinase domain, and truncation mutants. Hatched regions were deleted. (**b**) HEK-293A cells were co-transfected with plasmids expressing HA-tagged UL36 and Flag-tagged hMLKL truncation mutants. HA-UL36 was immunoprecipitated, and co-precipitating Flag-MLKLs were detected by Western blot. Flag-tagged IFI16 served as a negative control. (**c**) HEK-293A cells were transfected with plasmids as in (**b**). Flag-MLKLs were immunoprecipitated, and co-precipitating HA-UL36 was detected by Western blot. (**d**) Schematic depiction of mMLKL truncation mutants. (**e**) HEK-293A cells were co-transfected with plasmids expressing HA-UL36 and Flag-mMLKL truncation mutants. HA-UL36 was immunoprecipitated, and co-precipitating Flag-MLKLs were detected by Western blot. Flag-IFI16 served as a negative control. (**f**) HEK-293A cells were transfected with plasmids as in (**e**). Flag-MLKLs were immunoprecipitated, and co-precipitating HA-UL36 was detected by Western blot. + indicates transfection with pcDNA HA-UL36.

MLKL is composed of an N-terminal four-helix bundle domain, which enables MLKL to form oligomers and translocate to the plasma membrane, and a C-terminal pseudokinase domain containing the phosphorylation site [50]. Upon induction of necroptosis, RIPK3 phosphorylates MLKL, triggering the conversion of the inactive cytoplasmic MLKL protein to an oligomeric form that translocates to the plasma membrane and causes its permeabilization. Both N-terminal and C-terminal domains are essential for MLKL to elicit its function [51,52,53,54]. As it is unknown to which part of MLKL UL36 binds, we generated Flag-tagged mMLKL and hMLKL truncation mutants (Figure 3a,d).

HEK-293A were co-transfected with HA-UL36 and MLKL expression plasmids, and HA or Flag-tagged proteins were immunoprecipitated. Co-precipitating proteins were detected by immunoblot analysis (Figure 3b,c,e,f). UL36 co-precipitated with full-length hMLKL and the truncation mutants 100–472, but not with the other truncation mutants, indicating that the first 100 amino acids are dispensable for the interaction (Figure 3b,c). The N-terminal part of hMLKL (1–190) did not co-precipitate with UL36, suggesting that hMLKL amino acid residues 191 and 199 are needed for the interaction with UL36. By contrast, UL36 co-precipitated with full-length mMLKL and with the truncation mutants 100–465 and 200–465, but not with 300–465, indicating that its interaction with UL36 depends on a domain between amino acids 201 and 299 (Figure 3e,f). These data suggest that UL36 interacts with different domains of human and murine MLKL, respectively.

Interestingly, the MLKL truncation mutants lacking the entire N-terminal four-helix bundle were expressed at much lower levels in transfected cells (Figure 3) as compared to the full-length protein, suggesting that these truncated proteins are less stable.

### 3.3. UL36 Has a Higher Affinity to hMLKL Than to mMLKL

**Figure 4:**
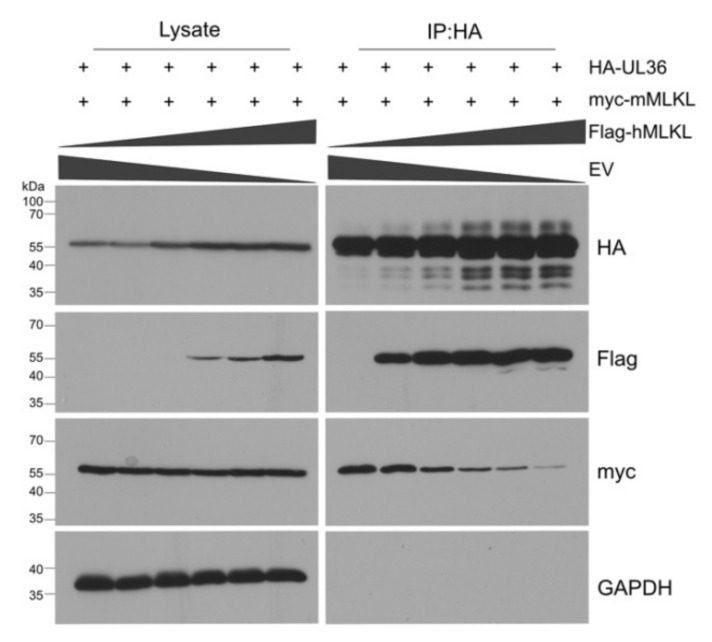
HCMV UL36 has stronger affinity to human than to murine MLKL. HEK-293A cells were co-transfected with 2.6 µg of plasmid expressing HA-tagged UL36, 2.6 µg of plasmids expressing myc-tagged mMLKL and increasing concentration (from 0 µg to 2.6 µg, black triangles) of plasmid expressing Flag-tagged hMLKL. HA-tagged UL36 was immunoprecipitated, and co-precipitating myc-tagged mMLKL and Flag-tagged hMLKL were detected by Western blot. Empty vector (EV) plasmid was used to normalize the total amount of transfected DNA. + indicates transfection with HA-UL36 and myc-mMLKL plasmids, respectively.

The results shown in Figure 2 and Figure 3 suggested that UL36 might interact more strongly with hMLKL than with mMLKL. To test whether UL36 has a higher affinity to hMLKL, we performed a competitive binding and immunoprecipitation assay. HEK-293A cells were co-transfected with constant amounts of HA-UL36 myc-mMLKL plasmids and increasing amounts of a Flag-hMLKL plasmid. HA-UL36 was immunoprecipitated and co-precipitating MLKL proteins were detected by immunoblot (Figure 4).

Under these conditions, even small amounts of hMLKL bound strongly to UL36, and hMLKL outcompeted mMLKL for binding to UL36. Interestingly, the steady-state levels of UL36 increased in both lysates and IP samples when hMLKL was expressed at higher concentrations. These data indicate that UL36 has a stronger affinity to hMLKL than mMLKL. They also suggest that UL36 levels are increased in the presence of hMLKL.

### 3.4. UL36 Promotes Proteasomal Degradation of mMLKL in Infected Cells

**Figure 5:**
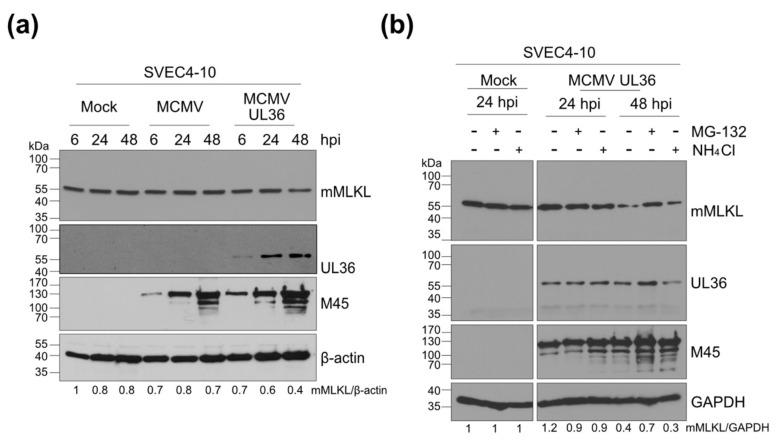
UL36 expression reduces mMLKL levels in MCMV-infected cells. (**a**) SVEC4-10 cells were infected with MCMV WT or MCMV-UL36. mMLKL, UL36, and M45 (infection control) levels were determined at different hours post-infection (hpi) by Western blot. (**b**) SVEC4-10 cells were infected with MCMV-UL36. Six hours before harvest, cells were treated with a lysosomal acidification inhibitor (NH_4_Cl) or a proteasome inhibitor (MG-132) or left untreated. mMLKL, UL36, and M45 levels were determined by immunoblot at 24 and 48 hpi. mMLKL levels relative to those of a housekeeping gene product were determined by densitometry and normalized to mock infected cells. + indicates treatment and – lack of treatment with the respective inhibitor.

UL36 has been shown to bind and degrade hMLKL in a proteasome-dependent manner [24]. To test whether UL36 does the same with mMLKL in murine cells, murine SVEC4-10 endothelial cells were infected at an MOI of 5 with a chimeric MCMV expressing UL36 instead of M36 (MCMV-UL36) [40]. The expression of UL36 in the MCMV-UL36 infected cells resulted in a modest reduction of mMLKL levels at 48 h post-infection (hpi) (Figure 5a).

To test whether the reduced mMLKL levels were a result of proteasomal or lysosomal degradation, we treated infected cells for 6 h before harvest with a proteasome inhibitor (MG-132) or a lysosomal inhibitor (NH_4_Cl). At 48 hpi, mMLKL levels were reduced in untreated cells and cells treated with NH_4_Cl, but were largely unchanged in cells treated with MG-132 (Figure 5b). These data suggested that UL36 promotes proteasomal degradation of mMLKL, similar to what has been described for UL36 in HCMV-infected human cells [24]. However, the reduction of mMLKL in cells infected with MCMVUL36 was quite modest, with substantial amounts of mMLKL remaining within infected cells.

### 3.5. A Chimeric MCMV-UL36 Virus Prevents Necroptosis in Humans but Not in Murine Cells

**Figure 6:**
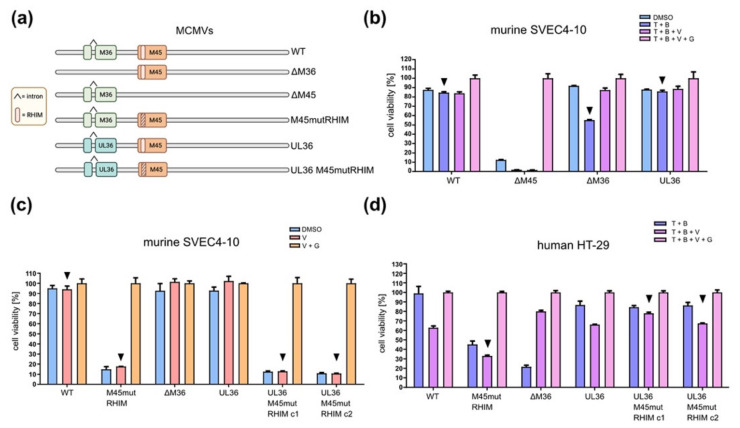
UL36 inhibits necroptosis in human but not in murine cells. (**a**) Schematic of MCMV mutants, generated with BioRender (accessed on September 1, 2021). (**b**) SVEC4-10 cells were infected with MCMVs (MOI 5) and treated 6 hpi with TNFα (T, 30 ng/mL), the SMAC mimetic BV6 (B, 1 µM), the caspase inhibitor zVAD-fmk (V, 75 µM), and the RIPK3 inhibitor GSK’872 (3 µM). Cell viability was determined by measuring ATP levels at 24 hpi. Values were normalized by group to cells treated with both inhibitors (T+B+V+G). (**c**) SVEC4-10 cells were treated with a caspase inhibitor (zVAD-fmk, 50 µM), a RIPK3 inhibitor (GSK’872, 5 µM), or vector (DMSO, 0.1%), and infected 1 h post-treatment with MCMV mutants. Cell viability was determined by measuring ATP levels at 24 hpi. For MCMV-UL36-M45*mut*RHIM two independent clones were used. Values were normalized by group to V+G treated cells (**d**) HT-29 cells were MCMVs infected and treated 6 hpi as in B. Cell viability was determined by measuring ATP levels at 24 hpi. Values were normalized by group to T+B+V+G-treated cells. For MCMV-UL36-M45*mut*RHIM two independent clones were used. Arrowheads indicate the samples to be compared.

UL36 can prevent caspase-8-dependent apoptosis and MLKL-dependent necroptosis in human cells [23,24]. A previous study has used the chimeric virus MCMV-UL36 to shown that UL36 can substitute for M36 in MCMV for the prevention of caspase-8-dependent apoptosis in infected cells [40]. Since UL36 interacts with mMLKL (Figure 2a,b) and reduces mMLKL levels (Figure 5), we interrogated whether UL36 also inhibits mMLKL-dependent necroptosis in murine cells. We first tested whether MCMV-UL36 can block apoptosis in SVEC4-10 endothelial cells. This cell line is highly sensitive to MCMV-induced necroptosis [27,55], but apoptosis induction requires appropriate stimulation, e.g., with TNFα and the Smac mimetic BV6 (T + B) [28,56]. As T + B treatment can induce both apoptosis and necroptosis, we used the caspase inhibitor z-VAD-fmk (V) to block apoptosis and promote necroptosis. The RIPK3 inhibitor GSK’872 (G) was used to block necroptosis. First, we used a set of MCMV mutants (Figure 6a) to confirm the ability of UL36 to inhibit TNFα-induced apoptosis in MCMV-infected cells.

Murine SVEC4-10 infected with MCMVΔM36 showed a significant reduction (*p* < 0.001) in cell viability when treated with T + B for apoptosis induction (Figure 6b). Apoptosis was inhibited by zVAD-fmk treatment (T + B + V). Cells infected with MCMV-UL36 were resistant to apoptosis induction (Figure 6b), indicating that UL36 functionally replaces M36 and prevents apoptosis in murine endothelial cells. As expected, SVEC4-10 cells were highly sensitive to infection-induced necroptosis when infected with an MCMV mutant lacking the viral necroptosis inhibitor, M45 (MCMVΔM45). Necroptosis could be inhibited with GSK’872 (Figure 6b).

Next, we wanted to find out whether UL36 can prevent necroptosis of murine cells. As MCMV already encodes a potent inhibitor of necroptosis, M45, whose activity depends on a RIP homotypic interaction motif (RHIM), we used a recombinant MCMV with a mutant RHIM (MCMV-M45*mut*RHIM). Murine SVEC4-10 cells infected with MCMV-M45*mut*RHIM lost viability due to necroptosis, as confirmed by treatment with necroptosis inhibitor GSK’872 (Figure 6c). When we mutated the M45 RHIM in MCMV-UL36, infected SVEC4-10 endothelial cells remained sensitive to necroptosis (Figure 6c). Similar results were obtained with MCMV-infected 10.1 fibroblasts (Supplementary Figure S1). These findings suggested that UL36 is unable to prevent necroptosis in murine cells. However, it remained possible that UL36 expression levels were insufficient. To exclude this possibility, we infected human HT-29 cells with the same set of MCMV mutants. In general, human cells are not permissive for the full replication cycle of MCMV, but they allow the expression of viral immediate-early and early gene products [57,58,59]. HT-29 cells are less sensitive than SVEC4-10 cells to infection-induced necroptosis, and therefore external stimulation (T + B) was used to induce apoptosis or necroptosis. As expected, HT-29 cells infected with MCMV-M45*mut*RHIM were sensitive to necroptosis, while those infected with MCMVΔM36 were sensitive to apoptosis (Figure 6d). When M36 was substituted by UL36 (MCMV-UL36), infected cells remained resistant to apoptosis induction, confirming that UL36 can rescue the loss of M36. Moreover, UL36 also rescued the loss of M45′s anti-necroptotic function (M45*mut*RHIM) in infected HT-29 cells (Figure 6d), indicating that UL36 expression by MCMV-UL36 is sufficient for the inhibition of necroptosis in human cells.

Taken together, the results of this study indicate that the HCMV UL36 protein is a species-specific necroptosis inhibitor. While UL36 interacts strongly with hMLKL (Figure 4) and is fully capable of inhibiting necroptosis in human cells (Figure 6d), consistent with recently published data [24], its interaction with mMLKL is less pronounced (Figure 4), and inhibition of necroptosis in murine cells was not detectable (Figure 6c).

## 4. Discussion

Herpesviruses have several proteins involved in escaping cellular antiviral defenses, and in particular in inhibiting programmed cell death [18,60]. In some cases, the inhibitory function of these proteins is species-specific, in other cases not. Here we show that the HCMV UL36 protein, which inhibits necroptosis in human cells, is incapable of preventing necroptosis in murine cells. The fact that UL36 inhibited necroptosis in human dermal fibroblasts [24] and HT-29 epithelial cells (Figure 6d) but not in murine endothelial cells (Figure 6c) or fibroblasts (Supplementary Figure S1) argues for a species-specific rather than a cell type-specific effect.

UL36 has been shown to block apoptosis by inhibiting caspase-8 activation in both human and mouse cells [40]. A recent study demonstrated that UL36 also blocks necroptosis by targeting MLKL, an effector protein of necroptosis, and degrading it via the proteasome [24]. The finding that UL36 acts as a dual cell death pathway inhibitor was reminiscent of the HSV-1 protein ICP6, which also inhibits both apoptosis and necroptosis [19,20,21,22]. However, UL36 and ICP6 are unrelated proteins, and their mechanisms of necroptosis inhibition are different: while ICP6 prevents RHIM-dependent activation of RIPK3, UL36 interacts and degrades MLKL [24]. While HCMV and HSV-1 use a single protein to block both apoptosis and necroptosis, UL36 and ICP6 [19,23,24,40], respectively, MCMV uses two separate proteins for this purpose: M36, a homolog of UL36, and M45, a homolog of ICP6, to block apoptosis and necroptosis, respectively [25,26,27,28,61]. It is also interesting to note that UL45, the HCMV homolog of ICP6 and M45, does not possess a RHIM and does not have a known role in cell death inhibition [62,63]. UL45 also differs from M45 and ICP6 in that it does not possess a conserved induced protein aggregation motif (IPAM) [64]. Nevertheless, it shares one of M45′s functions, the ability to inhibit NF-κB activation. The mechanisms, however, are not identical. While M45 interacts with RIPK1 and NEMO and induces their aggregation and degradation by aggrephagy [64,65,66], UL45 cooperates with the viral deubiquitinase UL48 to inhibit RIPK1-dependent NF-κB signaling [67].

Since human and murine MLKL proteins are highly conserved (62% amino acid identity and 84% similarity) and have the same function, we interrogated whether UL36 retains the ability to block necroptosis in murine cells. MLKL is the terminal effector of necroptosis, and it is activated by RIPK3. RIPK3 phosphorylates MLKL thereby initiating its oligomerization and translocation to the plasma membrane. Oligomerized MLKL permeabilizes the membrane and leads to necrotic cell death [50]. Although human and murine MLKL have similar sequences and structures, their interaction with RIPK3s is species-dependent. For example, hRIPK3 does not bind mMLKL and vice versa [68]. The species specificity of the RIPK3-MLKL interaction is primarily determined by sequence differences in the phosphorylation sites and the flanking sequences around the phosphorylation sites in hRIPK3 and mRIPK3 [68]. UL36 is thought to interact with and inhibit the unphosphorylated pool of hMLKL [24], therefore, it was reasonable to assume that UL36 could interact with mMLKL, regardless of the differences between mMLKL and hMLKL. By co-immunoprecipitation and immunofluorescence experiments, we verified that UL36 co-precipitates and co-localizes with mMLKL. This interaction was specific because the murine homolog, M36, did not interact with mMLKL. However, the interaction was stronger when UL36 was co-transfected with hMLKL (Figure 2 and Figure 3). Indeed, whenever UL36 and hMLKL were expressed together, the expression levels of UL36 increased, suggesting that hMLKL stabilizes the viral protein. Furthermore, as soon as hMLKL was expressed together with UL36, the interaction levels between UL36 and the murine protein decreased while those with the human protein increased (Figure 4). When we analyzed the requirement of specific MLKL domains for the interaction with UL36, we found that it differed between human and murine MLKL, suggesting a possible different regulatory mechanism (Figure 3). While studying the role of UL36 in necroptosis during infection, using an MCMV chimera, we noted that mMLKL expression levels were slightly decreased and were restored in the presence of a proteasome inhibitor (Figure 5). However, this level of degradation was apparently not sufficient to prevent the necroptosis in murine cells. In fact, neither during TNFα-induced cell death nor in cells susceptible to MCMV-induced necroptosis was it possible to observe an effect of UL36 on necroptosis. In contrast, UL36 significantly reduced necrosis in MCMV-infected human cells (Figure 6). Based on these data we concluded that the interaction of UL36 with mMLKL is weak and insufficient to prevent MLKL-mediated necroptosis in murine cells.

Many members of the *Herpesviridae* family are highly species-specific. Some of them replicate only in cells of their own or a closely related species [69]. Others can replicate in cells of a foreign species but cannot establish a productive infection in a foreign host organism. Numerous different factors contribute to the species barrier. After entering a cell, viruses need to overcome many hurdles to ensure their replication, such as cell-intrinsic defense mechanisms and the innate immune response. To this end, viruses have evolved proteins to inhibit or evade cellular defenses. As a result of a long co-evolution over thousands or millions of years, some viruses have become so adapted to their host that they can efficiently undermine cellular defenses, such as programmed cell death, only in cells of their natural host species. This seems to be of particular relevance to necroptosis inhibitors: HSV-1 ICP6 and HSV-2 ICP10 inhibit necroptosis in human but not murine cells [19,20,22]. The same is true for HCMV UL36, as we show in the present study. Moreover, a similar species-specific effect was observed in the PCD inhibitor MC159 of Molluscum contagiosum virus, a human poxvirus [48]. Necroptosis inhibitors should therefore be considered as host range factors.

## Data Availability

Data is contained within the article or supplementary material. The datasets generated and analyzed during the course of this study are available from the corresponding author upon request without restrictions.

## Supplementary Materials

The following are available online at https://www.mdpi.com/article/10.3390/v13112134/s1, Figure S1: UL36 does not prevent necroptosis in murine fibroblasts.
