# Supplementary material for: Species-Specific Inhibition of Necroptosis by HCMV UL36
Source: Viruses. 2021 Oct 22;13(11):2134. doi: 10.3390/v13112134 (PMC8621378; doi:10.3390/v13112134)
Supplement: Supplementary file 1 [file viruses-13-02134-s001.zip › viruses-1409730-supplementary.pdf]

Supplementary Figure S1

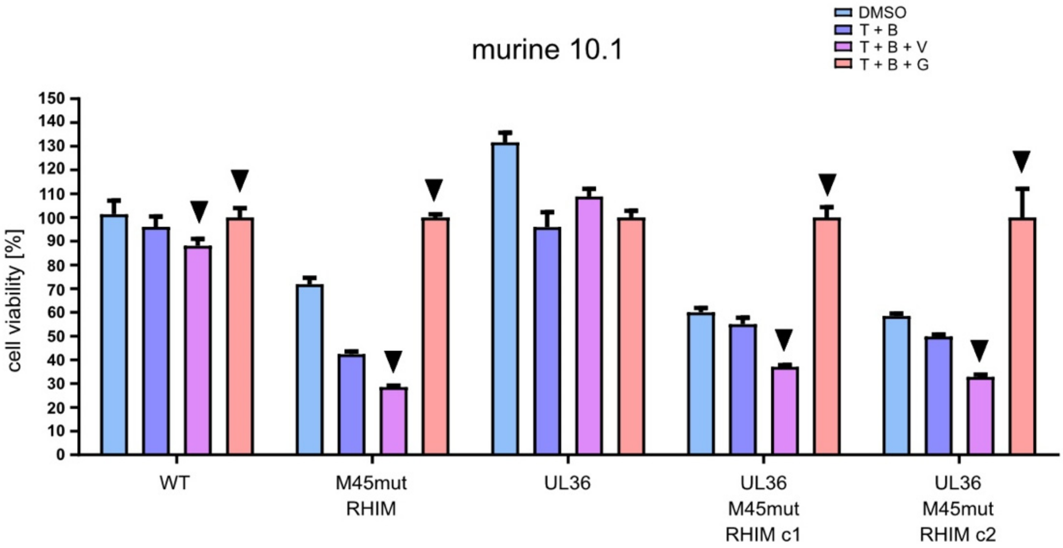

Supplementary Figure S1. UL36 does not prevent necroptosis in murine fibroblasts.
Murine 10.1 fibroblasts were infected with MCMVs (MOI 5) and treated 6 hpi with TNFα (T, 30 ng/mL), a SMAC mimetic (B, 1 μM), the caspase inhibitor zVAD-fmk (V, 75 μM), and the RIPK3 inhibitor GSK'872 (G, 3 μM). Cell viability was determined by measuring ATP levels at 24 hpi. Data are normalized to T+B+G. Black arrowheads indicate the samples to be compared.
